# Comparison of the Clinical Efficacy, Safety, and Postoperative Outcomes Between Peroral Esophageal Myotomy and Laparoscopic Heller's Myotomy With Fundoplication: A Systematic Review

**DOI:** 10.7759/cureus.44877

**Published:** 2023-09-07

**Authors:** Abishek Latha Kumar, Aishwarya Sadagopan, Anas Mahmoud, Maha Begg, Mawada Tarhuni, Monique N. Fotso, Natalie A Gonzalez, Raghavendra R Sanivarapu, Usama Osman, Tuheen Sankar Nath

**Affiliations:** 1 Internal Medicine and Pediatrics, California Institute of Behavioral Neurosciences & Psychology, Fairfield, USA; 2 Internal Medicine, California Institute of Behavioral Neurosciences & Psychology, Fairfield, USA; 3 Obstetrics and Gynecology, California Institute of Behavioral Neurosciences & Psychology, Fairfield, USA; 4 Pediatrics, California Institute of Behavioral Neurosciences & Psychology, Fairfield, USA; 5 Pulmonary and Critical Care Medicine, Texas Tech University Health Sciences Center, Midland and Odessa, USA; 6 Pulmonary and Critical Care Medicine, Nassau University Medical Center, East Meadow, USA; 7 Geriatrics, Michigan State University College of Human Medicine, East Lansing, USA; 8 Surgical Oncology, California Institute of Behavioral Neurosciences & Psychology, Fairfield, USA

**Keywords:** #esophageal achalasia, #heller myotomy, #peroral endoscopic myotomy (poem), #gastroesophageal reflux disease (gerd), #esophagitis

## Abstract

Achalasia, a neurodegenerative disease caused by the progressive destruction of ganglion cells in the myenteric plexus, is accompanied by incomplete relaxation of the lower esophageal sphincter. Laparoscopic Heller's myotomy (LHM) coupled with fundoplication has been the gold standard procedure for achalasia. Peroral esophageal myotomy (POEM) has recently gained popularity as it is minimally invasive, has fewer adverse events, and has excellent short-term outcomes. So, we aimed to compare the clinical efficacy, safety, and postoperative outcomes between LHM and POEM.

We did a systematic review by following the Preferred Reporting Items for Systematic Review and Meta-Analyses (PRISMA) guidelines for 2020 and exploring research databases such as PUBMED and PMC Central, Google Scholar, and Research Gate. After appropriate screenings, articles relevant to the review were scrutinized based on the eligibility criteria. Quality assessment tools such as the Newcastle-Ottawa Scale (NOS) and the assessment of multiple systematic reviews (AMSTAR) were used to finalize the articles. A total of 11 articles (seven observational studies, two RCTs, and two systematic reviews) were included in the review after a quality check. The study included 2127 patients, classified into 981 for POEM and 1146 for LHM, who had undergone treatment for achalasia. Most of the studies had a follow-up of ≤ two years.

Comparing efficacy, POEM had similar results to LHM in terms of Eckardt scores. However, abnormal DeMeester scores were found in POEM. Adverse events were significantly higher in LHM when compared to POEM in terms of safety. Peroral esophageal myotomy also stood out as having a shorter procedure time, a shorter hospital stay, and lesser odds of being a clinical failure. As for postoperative outcomes, despite treatment with proton pump inhibitors, LHM was more effective in preventing the development of esophagitis compared to POEM due to partial fundoplication.* *Postoperative reflux and the development of esophagitis remain certain with POEM and need to be followed up with more studies with longer follow-ups. However, POEM still stands as a better choice compared to LHM in terms of efficacy and safety.

## Introduction and background

Achalasia is an uncommon and rare esophageal motility disorder resulting from the loss of inhibitory neurons from progressive destruction and degeneration of the myenteric plexus, thereby leading to impaired relaxation of the lower esophageal sphincter (LES) and esophageal aperistalsis [1]. The incidence rate of 0.03 to 1.63 per 100,000 persons per year has recently increased to 2.3 to 2.93 cases per 100,000 [2]. According to Eckardt's score, patients usually present with progressive dysphagia of liquids and solids, retrosternal pain, regurgitation, and weight loss. The Chicago classification describes the types of achalasia for diagnosis and differentiation based on high-resolution esophageal manometry (HREM) [1]. There is no permanent cure for achalasia. Mild symptoms in elderly women were managed with nitrates and calcium channel blockers. Botulinum toxin injections (BTI) are reserved for candidates unsuitable for effective therapies [3]. Current treatment modalities are aimed at palliating symptoms, such as pneumatic dilation (PD) and laparoscopic Heller's myotomy (LHM) with fundoplication being the gold standard.

Heller's myotomy, first described in 1913 by German surgeon Ernst Heller, was modified in 1991 with the advent of the laparoscopic technique and became the gold standard with better long-term outcomes and fewer retreatments compared to PD [4]. Recently, an emerging surgical technique called peroral esophageal myotomy (POEM), a type of natural orifice endoscopic surgery, has gained popularity after Inoue et al. performed the procedure on 17 patients in 2008 [5]. It combines the advantages of being minimally invasive, like endoscopic dilation, with the precision of a surgical myotomy [6]. However, a suggested risk of POEM centers on the lack of fundoplication, which raises concern for the development of gastroesophageal reflux disease (GERD). Short-term outcomes have been excellent irrespective of age, type of achalasia, and prior interventions [7,8].

To date, very few studies have demonstrated the safety and clinical efficacy by comparing the two procedures and found the results to be similar. Therefore, this systematic review aimed to compare and analyze clinical efficacy, safety, and postoperative outcomes with adequate follow-up.

## Review

Methods

Search Strategy

This systematic review was planned and performed using the Preferred Reporting Items for Systematic Review and Meta-Analyses guidelines (PRISMA) 2020 to report the results [9]. A comprehensive search of published literature across MEDLINE, PubMed, PMC, Google Scholar, and Research Gate was conducted.

For MEDLINE, PubMed, and PMC, Medical Subject Heading (MeSH) was used to screen appropriate keywords pertaining to the topic and systematically identify relevant full-text articles [10-13]. The MeSH strategy included the following: Achalasia OR Impaired LES relaxation OR ( "Esophageal Achalasia/mortality"[Mesh] OR "Esophageal Achalasia/surgery"[Mesh] OR "Esophageal Achalasia/therapy“) AND Laparoscopic Heller myotomy OR Surgical Myotomy OR ( "Heller Myotomy/adverse effects"[Mesh] OR "Heller Myotomy/mortality"[Mesh] OR "Heller Myotomy/standards"[Mesh] OR "Heller Myotomy/statistics and numerical data"[Mesh] ) AND POEM OR Peroral Endoscopic Myotomy OR Natural Orifice Endoscopic surgery ("Natural Orifice Endoscopic Surgery/adverse effects"[Mesh] OR "Natural Orifice Endoscopic Surgery/mortality"[Mesh] OR "Natural Orifice Endoscopic Surgery/standards"[Mesh] OR "Natural Orifice Endoscopic Surgery/statistics and numerical data"[Mesh]). The keywords used for Google Scholar and Research Gate were as follows: “surgical myotomy”, "Hellers myotomy”, “peroral endoscopic myotomy”, "natural endoscopic orifice surgery”, “adverse effects”, “clinical efficacy”, “safety”, “postoperative outcomes”. The Booleans “AND,” “OR”, and “NOT” were used in multiple varying combinations along with keywords to find the relevant articles.

Inclusion and Exclusion Criteria

Observational studies, systematic reviews, and randomized clinical trials (RCT) published in English in the past 10 years were included. Exclusion criteria included articles pertaining to the pediatric population, grey literature, editorials, and animal studies. The full inclusion and exclusion criteria are listed in Table 1.

**Table 1:** Inclusion and exclusion criteria LHM: Laparoscopic Heller's myotomy, POEM: Peroral endoscopic myotomy

Inclusion criteria	Exclusion criteria
Articles from the last 10 years	Articles published before 10 years
English literature	Non-English literature
Human studies	Animal studies
Adult Study population	Pediatric study population
LHM	Robotic Heller's Myotomy, LHM without fundoplication
POEM	Pneumatic dilations, Botulinum toxin injections
Randomized clinical trials, observational studies, Systematic reviews, and meta-analyses	Case reports, editorials, letters, and articles not pertaining to the research question

Qualitative Analysis

A critical evaluation of articles was done for quality check and included 11 articles for final review. Quality assessment tools such as the Newcastle-Ottawa scale (NOS) for observational studies and assessment of multiple systematic reviews (AMSTAR) for systematic reviews and meta-analyses were used. Studies were assessed and graded based on the above quality assessment tools and only those that fulfilled as high quality were included in the final study. A comprehensive table depicting the quality converted according to standards (good, fair, and poor) is given below in Table 2.

**Table 2:** Newcastle-Ottawa scale scores for observational studies

Selection	Sanaka et al. [7]	Hungness et al. [14]	Kahaleh et al. [15]	Kumbhari et al. [16]	Sanaka et al. [17]	de Pascale et al. [18]	Schneider et al. [19]
Representativeness of the cohort	1	1	1	1	1	1	1
Selection of the non-exposed cohort	1	1	1	1	1	1	1
Ascertainment of exposure	1	1	0	1	0	1	1
Demonstration that outcome of interest was not present at the start of the study	1	1	1	1	1	1	1
Comparability							
Study controls for age (in this study)	1	1	1	1	0	1	1
Study controls for other factors	0	1	1	1	1	1	1
Outcome							
Assessment of outcome	1	1	1	1	1	1	1
Was follow-up long enough for outcomes to occur?	1	1	1	1	1	1	1
Adequacy of the follow-up cohorts	1	1	1	1	1	1	1
Total score	8/9	9/9	8/9	9/9	7/9	9/9	9/9
Quality grade	Good	Good	Good	Good	Fair	Good	Good

The randomized clinical trials [20,21] were assessed using the Cochrane risk of bias (RoB) assessment. Table 3 depicts the quality of systematic reviews.

**Table 3:** AMSTAR checklist for systematic reviews AMSTAR: Assessment of multiple systematic reviews; PICO: Patient/population, intervention, comparison, and outcomes; Rob: Risk of bias

AMSTAR criteria (Yes, Partial Yes, No)	Marano et al. [22]	Dirks et al. [23]
Did the research questions and inclusion criteria for the review include the components of PICO?	Yes	Yes
Did the report of the review contain an explicit statement that the review methods were established to conduct the review and did the report justify any significant deviations from the protocol?	Yes	Yes
Did the review authors explain their selection of the study designs for inclusion in their review?	No	Yes
Did the review authors use a comprehensive literature search strategy?	Partial Yes	Yes
Did the review authors perform study selection in duplicate?	Yes	Yes
Did the review authors perform data extraction in duplicate?	Yes	Yes
Did the review authors provide a list of excluded studies and justify the exclusions?	Partial Yes	Yes
Did the review authors describe the included studies in adequate detail?	Yes	Yes
Did the review authors use a satisfactory technique for assessing the RoB in individual studies that were included in the review?	Partial Yes	Yes
Did the review authors report on the sources of funding included in the review?	No	Yes
If meta-analysis was performed did the review authors use appropriate methods for statistical combination of results?	Yes	Yes
If meta-analysis was performed, did the review authors assess the potential impact of RoB in individual studies on the results of the meta-analyses or other evidence synthesis?	Yes	Yes
Did the review authors account for RoB in individual studies when interpreting/discussing the results of the review?	Yes	Yes
Did the review authors provide a satisfactory explanation for, and discussion of, any heterogeneity observed in the results of the review?	Yes	Yes
If they performed quantitative synthesis did review authors carry out an adequate investigation of publication bias (small study bias) and discuss its likely impact on the results of the review?	Yes	No
Did the review authors report any potential sources of conflict of interest, including any funding they received for conducting the review?	Yes	Yes
Total score	13/16 (High Quality)	15/16 (High Quality)

Data Extraction

Two investigators independently reviewed and extracted the data for all eligible studies related to the topic and screened them using the inclusion and exclusion criteria. Studies were assessed based on Eckardt scores, esophageal function for efficacy, adverse events and complications for safety, and the development of esophagitis in postoperative outcomes. All disagreements were resolved between the reviewers by reaching a consensus.

Results

**Figure 1:**
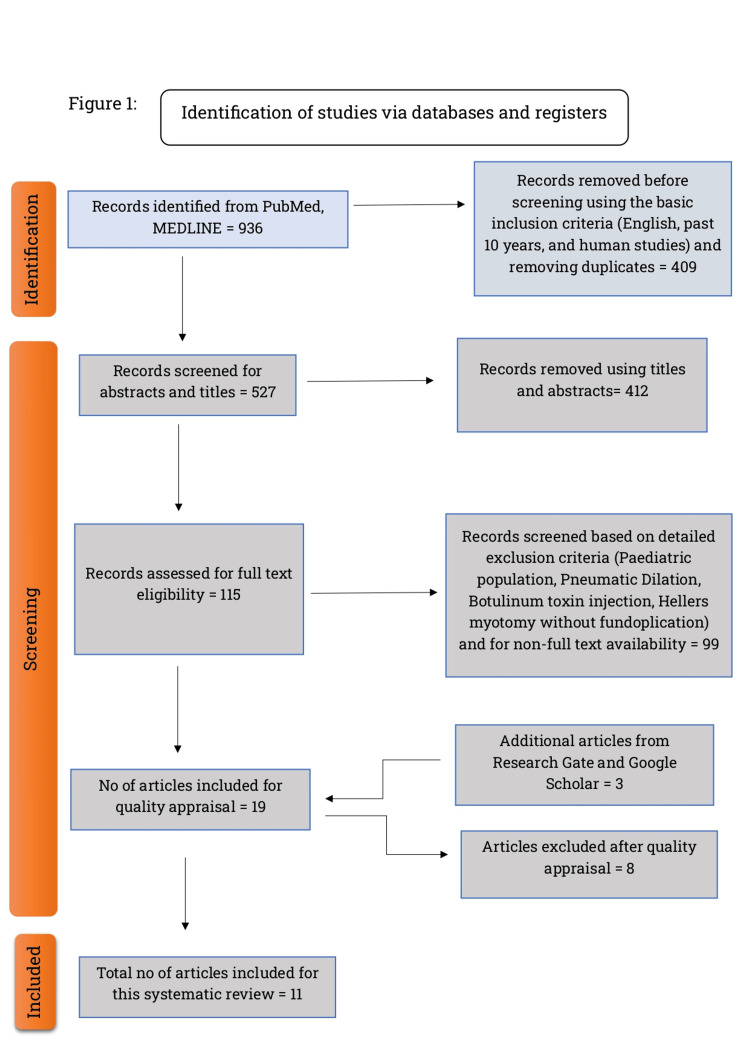
PRISMA 2020 flow diagram for systematic review

Using the appropriate MeSH strategy, 936 articles were found in PMC and PubMed. These included systematic reviews, RCTs, and observational studies. A total of 774 articles remained after filtering them using the basic exclusion criteria (non-English articles published before the past 10 years and animal studies). Around 527 articles were left after removing the duplicates. Articles were then carefully screened using abstracts, full-text papers, and detailed inclusion and exclusion criteria (Table 2) by two different authors to reduce the risk of selection bias. Two articles from Research Gate and one article from Google Scholar were then added that were relevant to our review. Eventually, 19 articles underwent quality appraisal using the above checklists. Eight articles were excluded after the quality check, leading to 11 articles (seven observational studies, two RCTs, and two systematic reviews) being included in the final review. A detailed flowchart using PRISMA 2020 is given below in Figure 1 [9].

The 11 articles that were finalized for this review included seven observational studies, two RCTs, and two systematic reviews. A total of 2127 patients had undergone treatment for achalasia which includes 981 for POEM and 1146 for LHM. A detailed description of the characteristics of the included studies except for systematic reviews is listed below in Table 4.

**Table 4:** Characteristics of the included studies LHM: Laparoscopic Heller's myotomy, POEM: Peroral esophageal myotomy; PD: Pneumatic dilation; RCT: Randomized clinical trial

Study	Years involved	Country of origin	Type of study	Interventions performed	Sample size	Follow-up
Sanaka et al. [7]	2014 - 2015	USA	Retrospective cohort	POEM vs. LHM	31 vs. 88	2 months vs. 2 months
Hungness et al. [14]	2004 - 2012	USA	Prospective cohort	POEM vs. LHM	18 vs. 55	6 weeks vs. N/A
Kahaleh et al. [15]	2014 - 2018	Latin America	Prospective cohort	POEM vs. LHM	69 vs. 64	17 months vs. 23 months
Kumbhari et al. [16]	2000 - 2013	USA, China, Japan, Denmark	Retrospective cohort	POEM vs. LHM	49 vs. 26	9 months vs. 22 months
Sanaka et al. [17]	2012 - 2015	USA	Prospective cohort	POEM vs. LHM vs PD	36 vs. 142 vs. 22	2 months vs. 2 months vs. 2 months
de Pascale et al. [18]	2012 - 2015	Italy	Retrospective cohort	POEM vs. LHM	32 vs. 42	24 months vs. 27 months
Schneider et al. [19]	2004 - 2016	Sweden	Retrospective cohort	POEM vs.LHM	25 vs. 25	36 weeks vs. 158 weeks
Werner et al. [20]	2012 - 2015	Europe	RCT	POEM vs. LHM	112 vs. 109	2 years vs.2 years
de Moura et al. [21]	2017 - 2018	Brazil	RCT	POEM vs. LHM	20 vs. 20	12 months vs. 12 months

In this systematic review, we were able to compare the safety and efficacy of POEM and LHM in most studies. These compared the Eckardt score, quality of life (QoL) metrics, and timed barium esophagogram (TBE) in the efficacy spectrum. Studies that compared the postoperative reflux between the two techniques found that POEM had a greater propensity for the development of GERD using the esophageal pH study findings, as POEM was not usually coupled with fundoplication compared to LHM [7]. Statistically, no difference was found between POEM and LHM in terms of perioperative outcomes and esophageal function [14]. Peroral esophageal myotomy was associated with higher short-term clinical outcomes in subtype three, in patients with Chagas disease, and in patients from Latin America [15]. The two systematic reviews included observational studies different from the ones we included in this review.

Discussion

By comparing the treatment of achalasia with LHM and partial fundoplication as the gold standard and the use of POEM, studies have shown us the benefits of excellent short-term clinical outcomes with the latter [8]. However, there is a serious concern about the long-term GERD symptoms in patients who undergo POEM. The curative treatment modality for achalasia lies in relieving the resting pressure at the LES by mechanical disruption. These included PD, LHM, and the more recent POEM [1]. Since POEM allows for a longer myotomy, it has been proposed as a treatment for patients with achalasia type III [16]. Several studies have demonstrated the effectiveness, long-term clinical outcomes, and postoperative morbidity of LHM with fundoplication to have excellent results. In one such study done by Krishnamohan et al. on the long-term outcomes of patients undergoing LHM with fundoplication, 85% of the patients did not report any symptoms five years after surgery, with a success rate of 76.1% at the 10-year follow-up [24].

In a study done by Sanaka et al., the results showed that the rate of esophageal exposure was higher in POEM compared to LHM (48.4% vs. 13.6%; p < 0.001) and that GERD symptoms had no statistical difference between POEM and LHM with fundoplication [7]. An RCT done by de Moura et al. showed that the rate of reflux esophagitis in the POEM group at one, six, and 12 months of follow-up was significantly higher in contrast to the LHM group (p = 0.014, p = 0.001, p = 0.002, respectively) [21]. Assessing the efficacy using esophageal function post-treatment as a parameter, a study done by Sanaka et al. in 2016 compared the existing treatments for achalasia. It was concluded that there was no statistically significant difference between POEM, LHM, and PD at the two-month follow-up (p > 0.005) [17]. Therefore, the studies conducted to compare the safety, efficacy, and long-term outcomes are very limited in nature, and with that in mind, this systematic review was designed with the aim of comparing the above-mentioned analysis to determine whether POEM has an extra edge over LHM.

Assessment of Efficacy

All of the included studies assessed the efficacy spectrum between POEM and LHM in terms of Eckardt scores, dysphagia scores, esophageal function post-treatment, TBE, and HREM in varying combinations. Each of the 11 studies stood out in terms of demographics such as population, study sample, study design, and eligibility criteria.

A prospective cohort study conducted by Kahaleh et al. over five years in Latin America included 113 patients, divided into 69 for POEM and 64 for LHM. A total of 35 patients had Chagas disease, out of which 17 were in POEM and 18 in LHM. Eckardt score and HREM pre- and post-POEM have been evaluated at three-, nine-, and 12-month follow-ups. The preprocedural assessment for Eckardt's score was 8.72 vs. 7.4 in the POEM vs. LHM groups, respectively (p < 0.001). Post-procedure, it was reduced to 1.84 vs. 3.6 in POEM vs. LHM (p < 0.001), respectively, at a follow-up of 16 months vs. 22 months in POEM vs. LHM (p = 0.0001). The initial therapy success rate was relatively high, at 86% in POEM vs. 60% in LHM. The preprocedural LES pressure was similar in both groups and was reduced to 14.3 mmHg in POEM and 19.8 mmHg in the LHM group post-procedure (p = 0.00231). This study concluded that POEM had a higher rate of initial success and was beneficial in a specific population due to the longer myotomy that it offers [15].

Kumbhari et al. conducted a retrospective study for patients with achalasia type III who underwent POEM (n = 49) across the US, Asia, and Europe, which was compared to an LHM (n = 26) done at a single tertiary institution in the US, respectively. Patients were classified according to the Chicago classification criteria. The preprocedural Eckardt stage in LHM compared to POEM had a higher mean baseline (2.85 vs. 2.37, p = 0.001). Eckardt's symptom stage reduced to < 1 was statistically significant with POEM vs. LHM (98.0% vs. 80.8%, p < 0.001) for a follow-up of 8.6 vs. 21.5 months, respectively. Since the study was performed in different geographic locations under diverse expertise, a larger proportion of the LHM group had a higher baseline Eckardt stage, and the follow-up in the POEM group was comparatively shorter, which could have led to a favorable bias toward POEM [16].

A retrospective study by Sanaka et al. in 2016 compared patients between LHM (n = 142), POEM (n = 36), and PD (n = 22) in terms of esophageal function based on TBE and HREM findings pre- and post-treatment at two months. Most of the patients who underwent POEM and PD had undergone prior treatments compared to the LHM group. The results showed no significant difference in the pre-treatment TBE and HREM parameters in all treatment groups (p > 0.05). The TBE's parameters included barium column height, width, and volume remaining measured at both one- and five-minute intervals. Actual barium height at two-month follow-up decreased by 50% in LHM and 47% in POEM (p = not significant). The HREM was measured on the scale of basal LES pressure and integrated relaxation pressure (IRP), which was reduced to less than 10 mmHg in 72% of LHM and 73% of POEM patients (p =not significant). The study had its limitations in age, BMI, and prior treatments in the POEM group, along with the statistical difference [17].

A cohort study done in a retrospective fashion by Pascale et al. between 2012 and 2015 had 74 patients divided into 32 for POEM and 42 for LHM. Preoperative assessment included an Eckardt score > 3, barium swallow, HREM findings, and esophagogastroduodenoscopy (EGD), which confirmed the diagnosis. The two groups were matched for age, sex, and previous treatment for achalasia. The median preoperative Eckardt score was six for both groups (POEM = 5 to 12, LHM = 4 to 12). A follow-up at 23 and 26 months for POEM and LHM, respectively, showed the Eckardt score was reduced to one in both groups (p = 0.001). This study tells us that there is no significant difference between the groups. However, the study had no predefined criteria for selecting a procedure and included patients who had had prior interventions [18].

In a study done by Schneider et al., patients were matched according to the eligibility criteria, which included Eckardt score, dysphagia severity score, QoL in reflux and dyspepsia (QOLRAD), and GERD-health-related quality of life (GERD-HRQL) questionnaire. A total of 25 patients in the POEM and 25 in the LHM group were matched according to the study's criteria. The preoperative mean Eckardt score was 6.6 (+/- 2.3) in both groups. Eckardt score < 3 was 91% and 84% in the POEM and LHM groups, respectively (p = 0.444). The four metrics mentioned above also improved in both groups at a median follow-up of 158 weeks for LHM and 36 weeks for POEM [19].

An RCT conducted by Werner et al. from 2012 to 2015 had a total of 221 patients randomized into LHM with fundoplication (n = 109) and POEM (n = 112). Clinical success was observed in 83% of POEM patients and 82% of LHM patients at two years of follow-up. The esophageal function was assessed objectively by measurement of IRP using HREM, which showed no significant difference (95% CI, −2.26 to 0.76). The gastrointestinal QoL metrics between baseline and 24 months showed no difference as well (95% CI, −4.01 to 4.28). However, this study had a serious limitation since less than 50% of eligible patients participated in the study [20].

A meta-analysis done by Marano et al. included papers between 2005 and 2015 involving 486 patients (n = 196 for POEM and n = 290 for LHM), in which five studies reported no significant difference between POEM and LHM in terms of Eckardt score reduction (p < 0.001). Since significant heterogeneity was present among the studies, random effect analysis was done to reduce the heterogeneity. One of the limitations of the study was that the follow-up was not more than a year, was non-randomized, and included patients with previous esophageal interventions [22]. A comparative table depicting the studies comparing the efficacy of the treatments is given in Table 5.

**Table 5:** Comparative table assessing the efficacy of studies TBE: Timed barium esophagogram, HREM: High-resolution esophageal manometry, POEM: Peroral esophageal myotomy, LHM: Laparoscopic Heller's myotomy, LES: Lower esophageal sphincter, QOLRAD: Quality of life in reflux And dyspepsia, QoL: Quality of life, GERD: Gastroesophageal reflux disease, IRP: Integrated relaxation pressure, HRQOL: Health-related quality of life

Author	Interventions studied	Type of study	Results	Conclusion
Kahaleh et al. [15]	POEM vs. LHM	Prospective cohort	POEM and LHM had significant reductions in Eckardt scores (p < 0.00001) but higher initial success therapy was found in POEM (p < 0.013). Mean LES sphincter pressure of 39 mmHg reduced to 14.3 mmHg in POEM and 19.8 mmHg in LHM	This study demonstrated that POEM was found to have higher rates of initial success and better in treating patients with achalasia due to a concurrent Chagas disease.
Kumbhari et al. [16]	POEM vs. LHM	Retrospective cohort	The primary outcome was a reduction to the Eckardt stage	POEM had a higher propensity of reduction in the Eckardt stage compared to LHM but a higher mean baseline Eckardt stage in LHM and shorter follow-up in POEM must be considered.
Sanaka et al. [17]	POEM vs. LHM vs. PD	Retrospective cohort	TBE in terms of barium column height reduced to 50 % in LHM and 47% in POEM. HREM parameters in terms of basal LES and IRP decreased to 72% in LHM and 73% in POEM.	Both LHM and POEM showed significant improvement in TBE and HREM and found no significant difference between the treatments.
Pascale et al. [18]	POEM vs. LHM	Retrospective cohort	The median preoperative Eckardt score of six was reduced to one in both groups.	POEM was equally efficacious to LHM
Schneider et al. [19]	POEM vs. LHM	Retrospective cohort	Preoperative Eckardt score of 6.6 was reduced in 91% and 84% of the POEM and LHM groups. Dysphagia, QOLRAD, and GERD-HRQL improved in both groups.	POEM shows better short-term outcomes and Eckardt score reduction
Werner et al. [20]	POEM vs. LHM	Randomized clinical trial	82% of POEM and 81% of LHM had clinical success at two years follow-up. HREM and GERD-QoL metrics did not report any significant difference.	This study concluded no significant difference was found in efficacy.
Marano et al. [22]	POEM vs. LHM	Systematic review and meta-analysis	There was no difference between POEM and LHM in terms of reduction in Eckardt score (p = 0.217)	POEM had equal efficacy to LHM

Assessment of Safety

Sanaka et al. in 2019 compared abnormal esophageal acid exposure and DeMeester score by performing a 24-hour pH study two months post-treatment between 31 POEM patients and 88 LHM patients. Patients were matched according to age, gender, race, BMI, and duration of symptoms. The results of this study showed that abnormal esophageal acid exposure was greater in POEM vs. LHM patients (48.4% vs. 13.6%, p < 0.001) with an abnormal DeMeester score of 54.8% vs. 17.4%; p = 0.005). This subset of the POEM group had a greater number of prior achalasia interventions compared to the LHM group (overall: 71% vs. 44.3%; p = 0.012; PD: 35.5% vs. 18.2%; p = 0.041; LHM: 32.3% vs. 0%; p = 0.003, respectively). There was no significant difference between POEM and LHM in terms of GERD symptoms (28% vs. 15%, p = 0.38) [7].

A prospective cohort study done by Hungness et al. in 2012 compared the perioperative outcomes between LHM and POEM in a non-randomized fashion. It included 73 patients (18 in the POEM group, and 55 in the LHM group) who were matched, including the absence of prior treatments. The results showed that operative times were shorter for POEM (113 min vs. 125 min; p < 0.05) and the estimated blood loss was less compared to LHM (<10 ml vs. 50 ml; p < 0.001). Adverse events and hospital stays were similar [14]. The safety of the procedures was considered in terms of procedure time, adverse events, and peri- and post-operative complications. In the study done by Kumbhari et al. (49 in the POEM group, 26 in the LHM group), POEM had a significantly shorter mean procedure time compared to the LHM group (102 min vs. 264 min; p < 0.01). No significant difference was found in hospital stay (3.3 days vs. 3.2 days; p = 0.68), despite having a statistically significant myotomy in POEM vs. LHM (16cm vs. 8 cm; p < 0.01). The overall rate of adverse events was significantly higher in the LHM group (27% vs. 6%; p = 0.01). The adverse events were classified as moderate in seven of the LHM patients, compared to one-third of the patients in the POEM group [16].

In the study done by Kahaleh et al., the rate of adverse events in the POEM group was 17% compared to 14% in the LHM group (p = not significant). These included pneumothoraces, bleeding requiring transfusion, and mediastinitis. The LHM group had longer hospital stays compared to POEM (p < 0.00001) [15]. Adverse events in a study done by Werner et al. in 2019 involved 221 patients (112 in the POEM group and 109 in the LHM group), revealing 2.7% in the POEM group and 7.3% in the LHM group. Procedure time was found to be shorter in the POEM group than in the LHM group by 13 minutes (95% CI, 6.26 to 21.36), with no difference in the length of hospital stay [20]. No statistically significant difference was noted in the rate of adverse events in the RCT study done by de Moura et al. [21]. Dirks et al. conducted a systematic review and meta-analysis between POEM, LHM, and PD on the efficacy scale, perioperative metrics, and safety. The review included 21 studies comparing LHM and POEM, which showed no statistically significant difference in serious adverse events, complications, or hospital stays [23]. In a systematic review by Marano in 2016, there was no significant difference in operative time (p = 0.001) or hospital stay (p = 0.049). When comparing gastrointestinal reflux rates, LHM demonstrated better outcomes in contrast to POEM (95% CI: 1.11-1.95, p < 0.017) [22]. Table 6 depicts the safety measures in the studies.

**Table 6:** Table comparing the safety of the studies POEM: Peroral esophageal myotomy, LHM: Laparoscopic Heller's myotomy, PD: Pneumatic dilation, GERD: Gastroesophageal reflux disease, EBL: Estimated blood loss

Author	Interventions studied	Type of Study	Results	Conclusion
Sanaka et al. [7]	POEM vs. LHM	Retrospective cohort	POEM group had abnormally higher esophageal exposure than the LHM group (48% vs. 14%, p < 0.001). No significant difference was however found in GERD symptom rate.	This study concluded that POEM led to increased esophageal exposure compared to LHM but no significant difference existed in terms of GERD symptoms
Hungness et al. [14]	POEM vs. LHM	Prospective cohort	Operative times were shorter in POEM group vs. LHM (113 minutes vs. 125 minutes, p < 0.05). EBLs were significantly less in POEM compared to LHM. Perioperative adverse events were similar between the groups.	Although POEM had lesser procedure time and reduced EBLs, the advantage of these minuscule differences will translate into clinical benefits for patients.
Kahaleh et al. [15]	POEM vs. LHM	Retrospective cohort	Adverse events were noted in 17% of the POEM group (n = 69) and 14% of the LHM group (n = 64). Hospital stay was considerably longer in the LHM group (p < 0.000001)	LHM showed a rate of adverse events higher than POEM and in terms of hospital stay as well
Kumbhari et al. [16]	POEM vs. LHM	Retrospective cohort	Shorter procedure time in POEM was observed compared to LHM (102 minutes vs. 264 minutes; p < 0.01) despite a longer myotomy in POEM. The adverse event rate was statistically significant and higher in the LHM group (27% vs. 6%, p = 0.01).	This study in terms of safety concluded that POEM had fewer adverse events and procedure time.
Marano et al. [22]	POEM vs. LHM	Systematic review and meta-analysis	No significant results were found in terms of operative time (p = 0.36) and hospital stay(p = 0.049) between POEM and LHM. Overall minor and major complication rates also did not differ between the groups (p = 0.74, p = 0.3), respectively.	POEM had a similar safety profile to LHM for achalasia at a short-term follow-up
Dirks et al. [23]	POEM vs. LHM vs. PD	Systematic review and meta-analysis	This study had no difference in adverse events, return to the operating room for complications, or unexpected ICU stay.	POEM had similar safety outcomes to LHM and PD. But in terms of efficacy, POEM had an upper hand over PD.

Assessment of Postoperative Outcomes

When compared with POEM in existing literature and clinical practice, the LHM had better postoperative and long-term outcomes. Most of the studies in this review reported that POEM had an increased occurrence of esophagitis compared to LHM. In a study by de Pascale et al. that assessed endoscopy at the one-year follow-up, 20% in each group completed the morphological evaluation for endoscopy, whereas 40% (eight patients) in the POEM group compared to 5% (one patient) in the LHM group had esophagitis (p = 0.004, 95% CI) [18]. This esophagitis was classified using the Los Angeles classification.

**Figure 2:**
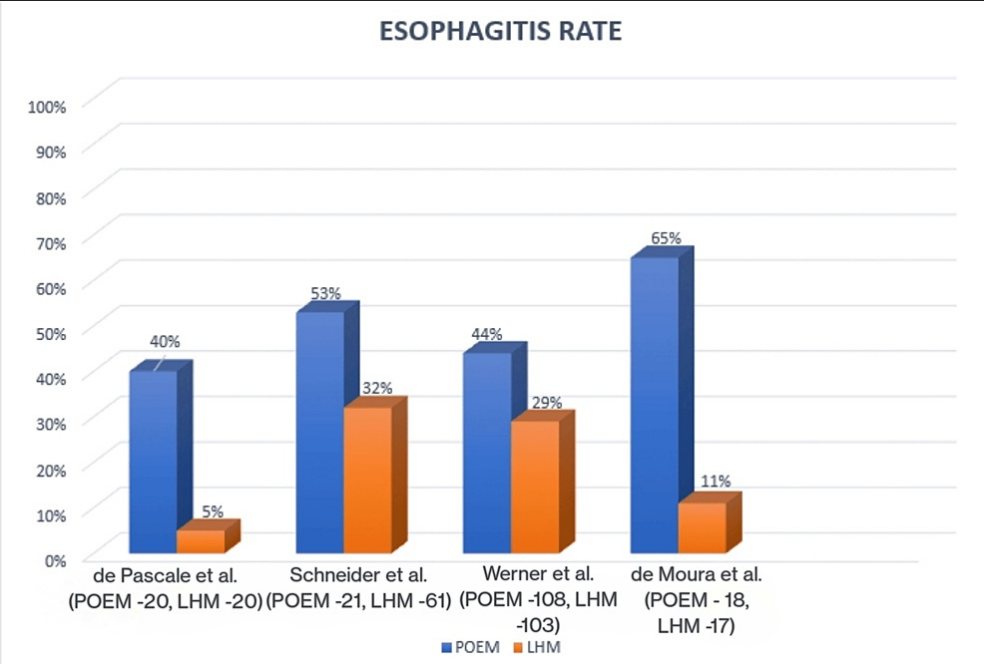
Graph showing the rate of esophagitis in different studies POEM: Peroral esophageal myotomy, LHM: Laparoscopic Heller's myotomy

Schneider et al. reported in their study that postoperative endoscopy was performed in 50% of POEM and 73% of LHM (p = 0.087), where esophagitis was noted to be 53% in POEM and 32% in LHM [19]. A study performed by Werner et al. involved 108 POEM patients and 103 LHM patients and assessed the development of GERD clinically, endoscopically, and through pH monitoring. The results showed us that esophageal reflux was higher in the POEM group compared to the LHM group at three months, which was 57% vs. 20% (odds ratio, 5.74; 95% CI, 2.99 to 11.00), and at 24 months, it was 44% vs. 29% (odds ratio, 2.00; 95% CI, 1.03 to 3.85) [20]. Another RCT done by de Moura et al. evaluated 40 patients by dividing them into groups of 20 each for POEM and LHM. At the one-, six-, and 12-month follow-ups, the rates of esophagitis were significantly higher in the POEM group compared to the LHM group (p = 0.014, p < 0.001, and p = 0.002, respectively). The rates of esophagitis were 0.0%, 5.6%, and 11.1% in the LHM group and 29.4%, 62.5%, and 64.6% in the POEM group at the one-, six-, and 12-month follow-up, respectively [21]. Marano et al. did a meta-analysis that concluded that POEM had worse short-term results in comparison to LHM concerning the development of post-procedure GERD [22]. Figure 2 represents the esophagitis rates of the above studies in a simpler format and represents only patients who were able to complete the endoscopic evaluation at their respective follow-up visits.

## Conclusions

This systematic review was developed to compare the clinical efficacy, safety, and long-term outcomes between POEM and LHM. In terms of efficacy, Eckardt score reduction pre- and post-procedure and QoL metrics were observed to be similar, with no statistical significance between both procedures. When it comes to safety, POEM has the upper hand in procedure time. However, the rate of adverse events and length of hospital stay were not significant. Postoperative outcomes, including esophagitis and the development of GERD, were noticed more in POEM patients than in LHM patients. Some studies suggest that the initial clinical success rate is relatively higher in POEM and that there is a better prognosis in treating patients with Chagas disease-affected achalasia and achalasia subtype III.

This review will guide future surgeons and physicians in training to keep these findings in mind before selecting a procedure based on patient preference. Even though LHM is coupled with fundoplication, POEM can be considered a safe option given its duration and low rate of adverse events. Long-term outcomes must be evaluated with more RCTs and a larger sample size to have a more complete and detailed validation.
